# Supplementary material for: Biodiverse food plants in the semiarid region of Brazil have unknown potential: A systematic review
Source: PLoS One. 2020 May 7;15(5):e0230936. doi: 10.1371/journal.pone.0230936 (PMC7205212; doi:10.1371/journal.pone.0230936)
Supplement: S1 File — (DOCX) [file pone.0230936.s002.docx]

File 2 Research strategy for systematic review

| SCOPUS (ELSEVIER) | TITLE ( food OR "FOOD PLANT" ) AND TITLE ( caatinga OR "DRY SEASONAL FOREST" OR "SEASONAL DRY FOREST" OR "SEASONAL TROPICAL DRY FOREST" OR "SEASONAL FOREST" OR "DRY FOREST" OR "TROPICAL DRY FOREST" ) AND brazil AND ( LIMIT-TO ( PUBYEAR , 2020 ) OR LIMIT-TO ( PUBYEAR , 2019 ) OR LIMIT-TO ( PUBYEAR , 2018 ) OR LIMIT-TO ( PUBYEAR , 2017 ) OR LIMIT-TO ( PUBYEAR , 2016 ) OR LIMIT-TO ( PUBYEAR , 2015 ) OR LIMIT-TO ( PUBYEAR , 2014 ) OR LIMIT-TO ( PUBYEAR , 2013 ) OR LIMIT-TO ( PUBYEAR , 2012 ) OR LIMIT-TO ( PUBYEAR , 2011 ) OR LIMIT-TO ( PUBYEAR , 2010 ) OR LIMIT-TO ( PUBYEAR , 2009 ) OR LIMIT-TO ( PUBYEAR , 2008 ) ) REFINE RESULTS: LIMIT TO (YEAR: 2008-2018) (DOCUMENT TYPE: ARTICLE) (LANGUAGE: ENGLISH, PORTUGUESE, SPANISH) |
| --- | --- |
| MEDLINE/ PubMed | ((FOOD OR "FOOD PLANT"[MESH TERMS])) AND ((CAATINGA OR "DRY SEASONAL FOREST" OR "SEASONAL DRY FOREST" OR "SEASONAL TROPICAL DRY FOREST" OR "SEASONAL FOREST" OR "DRY FOREST" OR "TROPICAL DRY FOREST"[MESH TERMS])) AND (BRAZIL[MESH TERMS])) SORT BY: PUBLICATION DATE FILTERS: PUBLICATION DATE FROM 2008/01/01 TO 2020/02/26 LANGUAGES: ENGLISH OR PORTUGUESE OR SPANISH |
| EMBRAPA - BDPA | ((ASSUNTO: PLANTAS AND CAATINGA) AND (TIPO-MATERIAL-SIGLA:"AP") AND (IDIOMA:"ESPANHOL" OR IDIOMA:"INGLÊS" OR IDIOMA:"PORTUGUÊS") AND (ANO-PUBLICACAO:[2008 TO 2020])) |
| WEB OF SCIENCE | (TS=(("FOOD" OR "FOOD PLANT") AND ("CAATINGA" OR "DRY SEASONAL FOREST" OR "SEASONAL DRY FOREST" OR "SEASONAL TROPICAL DRY FOREST" OR "SEASONAL FOREST" OR "DRY FOREST" OR "TROPICAL DRY FOREST") AND ("BRAZIL"))) AND DOCUMENT TYPES: (Article) Timespan: 2008-2020. |
